# A blueprint for global registries from Norway

**DOI:** 10.1080/17453674.2020.1763604

**Published:** 2020-05-14

**Authors:** Stephen Ellis Graves

In 2000 ACTA published a landmark article authored by the leads of the Norwegian Arthroplasty Registry. Reviewing it today it is easy to understand why it is one of the 10 most cited ACTA articles of the last 20 years.

The Norwegian Arthroplasty Registry commenced hip data collection in 1987 and expanded to include other arthroplasty procedures in 1994. In 2000 it was one of a small number of quality national arthroplasty registries with sufficient follow up to provide meaningful data. Others included the Swedish Knee, Swedish Hip and Finnish registries. Publications by these foundation registries particularly in the 10 or so years prior had brought world attention to the value of registry data. By the late 1990’s and early 2000’s many other national registries were being established or planned. The importance of this publication was that it was a blueprint for developing registries on how to successfully implement and manage a national arthroplasty registry.

The article describes the purpose, methodology, governance, and achievements of the Norwegian Arthroplasty Registry. It provided a rational for why registries should be established and how beneficial change could be achieved. Selective examples of registry analysis were also provided that highlighted the value and importance of this data.

Registries are quality assurance mechanisms designed to monitor and provide real time stakeholder feedback. Their purpose is to ensure continuous quality improvement. Assessing and reporting comparative prosthesis performance is one of those important quality assurance activities. The article justifies the need for this because at that time it was common practice for surgeons to implant “undocumented” prostheses i.e. prostheses without evidence. Regulators did not require prosthesis specific premarket clinical evaluation and post market clinical trials were rarely undertaken. A number of examples of registry identified outlier performance were provided including the Norwegian Registry “Boneloc” identification. Despite the litany of prosthesis specific problems reported by registries since that time disappointingly there has been little change to regulatory premarket evaluation requirements and the use of “undocumented” prostheses continues.

Performance also varies by prosthesis class. Evidence was presented on reduced revision rates in younger patients associated with some designs of cementless compared to cemented femoral stems. This and the identification of specific characteristics of cementless designs associated with success was important information at that time. Comments on the need to better evaluate metal on metal bearings and the potential good outcomes of crosslinked polyethylene were eerily predictive and reflect the informed commentary, expertise and understanding of the Norwegian Arthroplasty team.

Critical to registry success is surgeon engagement. A strategy recommended by the Norwegian Registry was the provision of regular reports to participating hospitals. Surgeons being informed of their comparative performance either personal or through practice hospital data is now known to be a major incentive for ongoing participation and consequently most registries provide this type of data. Successful engagement also requires surgeon confidence in data accuracy and completeness, analytical techniques as well as transparency and accountable governance. The article provided specific information on each of these issues.

It is only high-quality publications that are frequently cited. They clearly and succinctly define the issues and provide real and possible solutions, grow knowledge and provide unexpected insights. They often stand the test of time. This article has all of these characteristics and it is just as valuable and relevant today as when it was published 20 years ago.

Stephen Ellis Graves *Email:*segraves@aoanjrr.org.au

